# Development of a recombinant protein based dot-blot for the diagnosis of canine leptospirosis

**DOI:** 10.1186/1753-6561-8-S4-P144

**Published:** 2014-10-01

**Authors:** Neida Conra, André Grassmann, Rafaela Xavier, Caroline Gonçalves, Flavia McBride, Alan McBride

**Affiliations:** 1 Núcleo de Biotecnologia, CDTec, Universidade Federal de Pelotas, Pelotas, Brazil; 2 UniversidadeCatólica de Pelotas, Pelotas, Brazil

## Background

Leptospirosis is a globally disseminated zoonosis, recognized as a re-emergent neglected disease [1] and the causative pathogens are spirochetes from genus *Leptospira *[2]. In Brazil, more than 100.00 leptospirosis cases are reported annually, with outbreaks emerging especially during floods [3]. The microscopic agglutination test (MAT) is considered the standard serological test for the diagnosis of leptospirosis, but it requires paired serum samples, is demanding, difficult to analyse and the results can be variable when compared between laboratories [2]. The absence of an adequate laboratorial diagnosis is the main barrier for the implementation of disease surveillance for the control of both human and animal leptospirosis. Hence, there is an urgent need for the development of an adequate laboratorial test that is both quick and reliable. Apart from the danger connected to rodents, which are leptospires main vectors, occurrence of the disease in dogs can generate a higher risk of infection for humans [4]. In the current study, selected recombinant proteins from *L. interrogans *were tested for their diagnostic potential using a dot-blot technique against a canine serum panel previously characterized by the MAT and a whole-cell *Leptospira *indirect ELISA.

## Methods

Nine recombinant proteins were tested for their ability of recognize antibodies in Leptospirosis positive and negative serum samples. The proteins were based on LigB, LigA, OmpL37, LemA, FlaA1, FlaB1, and LipL32. Three different concentrations were evaluated per antigen: 300, 500 and 1000 ng in each dot-blot. Two pooled serum dilutions were tested: 1:200 and 1:500. Furthermore, two isotypes of conjugated antibodies were evaluated: anti-dog IgG and anti-dog IgM. Selected recombinant proteins were screened using a panel of individual canine serum samples: 19 positive (11 vaccinated and 8 non-vaccinated) and 19 negative (11 vaccinated and 8 non-vaccinated). The degree of reaction was classified in four levels of colour (-, +1, +2 and +3), where +2 and +3 were considered positives and - and +1 negatives.

## Results and conclusions

The majority of the tested antigens showed weak, non-specific reactions with the positive sera, except for rLigBrep and rLipL32. These recombinant proteins distinguished between the positive and negative pooled sera. Using anti-IgG, rLipL32 recognised all antigen concentrations at a 1:500 sera dilution. For rLigBrep, the best performance was achieved with anti-IgM and 500 ng and 1000 ng of antigen, regardless of the sera dilution. In an analysis of the individual serum samples, the optimal concentration of rLigBrep was 150 ng, and the sensitivity was 81.8% (9/11) and specificity was 63.6% (7/11). Using serum samples from vaccinated animals, the rLigBrep had a sensitivity of 87.5% (7/8) and specificity of 25% (2/8). rLipL32 failed to discriminate between the positive and negative sera and was not included in further evaluations. From these data, we can conclude that the dot-blot technique showed promising results in discriminating between positive and negative, vaccinated and non-vaccinated canine serum samples. However, further refinement of the technique is required before it can be used for the diagnosis of canine leptospirosis.
